# Stromal cell extracellular vesicular cargo mediated regulation of breast cancer cell metastasis via ubiquitin conjugating enzyme E2 N pathway

**DOI:** 10.18632/oncotarget.22371

**Published:** 2017-11-10

**Authors:** Krishna C. Vallabhaneni, Patrice Penfornis, Fei Xing, Yoni Hassler, Kristen V. Adams, Yin-Yuan Mo, Kounosuke Watabe, Radhika Pochampally

**Affiliations:** ^1^ Cancer Institute, University of Mississippi Medical Center, Jackson, MS 39216, USA; ^2^ Department of Radiation Oncology, University of Mississippi Medical Center, Jackson, MS 39216, USA; ^3^ Department of Cancer Biology, Wake Forest University School of Medicine, Winston-Salem, NC 27157, USA; ^4^ Department of Pathology, University of Mississippi Medical Center, Jackson, MS 39216, USA; ^5^ Comprehensive Cancer Center, Wake Forest University School of Medicine, Winston-Salem, NC 27157, USA; ^6^ Department of Biochemistry, University of Mississippi Medical Center, Jackson, MS 39216, USA

**Keywords:** exosomes, dormancy, MSCs, miRNA, signalling

## Abstract

Mesenchymal stromal cells (hMSCs) have been used to understand the stromal cell properties in solid tumors because of their ablity to differentiate into most cell types. We investigated the role of EVs from hMSCs (hMSC-EVs) in breast cancer metastasis using MDA-MB-231 parental cell line and organotropic sub-lines. We demonstrated that hMSC-EVs significantly suppressed the metastatic potential of the parental cell line when compared to their organotropic sublines. hMSC-EVs induce dormancy in the parental cell line but not in their organotropic sub-lines and miR-205 and miR-31 from EV cargo played a role. Further, Ubiquitin Conjugating Enzyme E2 N (UBE2N/Ubc13) - metastasis-regulating gene, is a target of these miRNAs and silencing of UBE2N/Ubc13 expression significantly suppressed migration, invasion, and proliferation of breast cancer cells. To summarize, hMSC-EVs support primary breast tumor progression but suppress the metastasis of breast cancer cells that are not organ-committed through the UBE2N/Ubc13 pathway and play a role in premetastic niche formation.

## INTRODUCTION

Breast cancer is the most prevalent cancer in women and the second leading cause of death worldwide [1]. Although early detection and treatment has decreased the mortality of breast cancer, metastatic breast cancer accounts for approximately 40,000 deaths annually in the U.S. [2–4]. Successful treatment of metastatic breast cancer is difficult without greater knowledge of the underlying molecular mechanisms of metastasis, which include interactions between tumor microenvironment and cancer cells. Within the tumor microenvironment are stromal cells with mesenchymal stem/stromal cells (hMSCs) like phenotype, which play a major role and have been shown to exert effects through the release of tropic factors and extracellular vesicles (EVs) [5–9]. The EVs, ranging in size from 30–100 nm in diameter, are also called exosomes and transport proteins, lipids, mRNA, miRNA, and small molecule metabolites. As such, these EVs are considered paracrine effectors, as they mimic the therapeutic effects of hMSCs in various diseases [10, 11]. Depending on the cancer type and the experimental models, EVs either promote or suppress tumor growth [12]. Identifying the molecular mechanisms that support cancer cell proliferation would improve our understanding of the dynamics of cell to cell communication within the tumor microenvironment.

Earlier studies in our lab using stressed hMSCs have shown the tumor-supporting properties of EVs and the of microRNAs (miRNAs) present in the cargo exert cell survival signals in the nutrient-deprived tumor microenvironment [13]. MicroRNAs, short non-coding RNAs that post-transcriptionally regulate gene expression, are differentially expressed in different types of cancers and are involved in the regulation of biological processes and genes that are part of the metastatic cascade. [14, 15]. In breast cancer, miR-17-5p, miR-125a, miR-125b, miR-200, miR-205, miR-206, let-7, miR-34, and miR-31 were identified as tumor suppressor genes, and miR-21, miR-155, miR-10b, miR-373, and miR-520c were found to be oncogenes [16–19]. Understanding the magnitude of EV-miRNA involvement in cancer metastasis is crucial for evaluating their therapeutic potential.

In this study, two *in vivo* mouse models were used to study the role of stressed hMSC-derived EVs (hMSC-EVs) in regulating the metastatic potential of breast tumors. First, a xenograft-metastatic mouse model was used to understand the role of hMSC-EVs in homing of cancer cells to various organs and their progression at those sites. Second, organotropic breast cancer sub-lines and an orthotopic mouse model were used to study the role of hMSC-EVs in primary tumor progression. hMSC-EVs induced dormancy in a sub- population of the parental cell line, inhibiting their proliferation and 3D sphere-forming ability. Further bioinformatics evaluation of EV-miRNA cargo to shortlist and focus on specific targets for their role in metastasis lead to selecting miR205 and 31 for further mechanistic studies. The expression of tumor suppressor miRNAs, miR-205 and miR-31, was upregulated when parental cancer cells were treated with hMSC-EVs. MiR-205 and miR-31 specifically blocked the metastatic ability of breast cancer cells by modulating Ubiquitin Conjugating Enzyme E2 N (UBE2N/Ubc13), which is a common target gene for these miRNAs and is known to be involved in breast cancer metastasis. Loss of UBE2N/Ubc13 inhibited breast cancer cell proliferation, migration, and invasion, presumably increasing dormancy. Our findings indicate that stressed hMSC-EVs support primary breast tumor growth but suppress the metastasis of non-organ-committed breast cancer cells (MDA-MB-231/231 cells) compared with their organotropic sub-lines (231BrM-2a, 231LM-4175, and 231BM-1833).

## RESULTS

### hMSC-EVs supresses metastasis of MDA-MB-231 non-specific metastasis cells but not in organ specific metastasis cells

**Figure 1:**
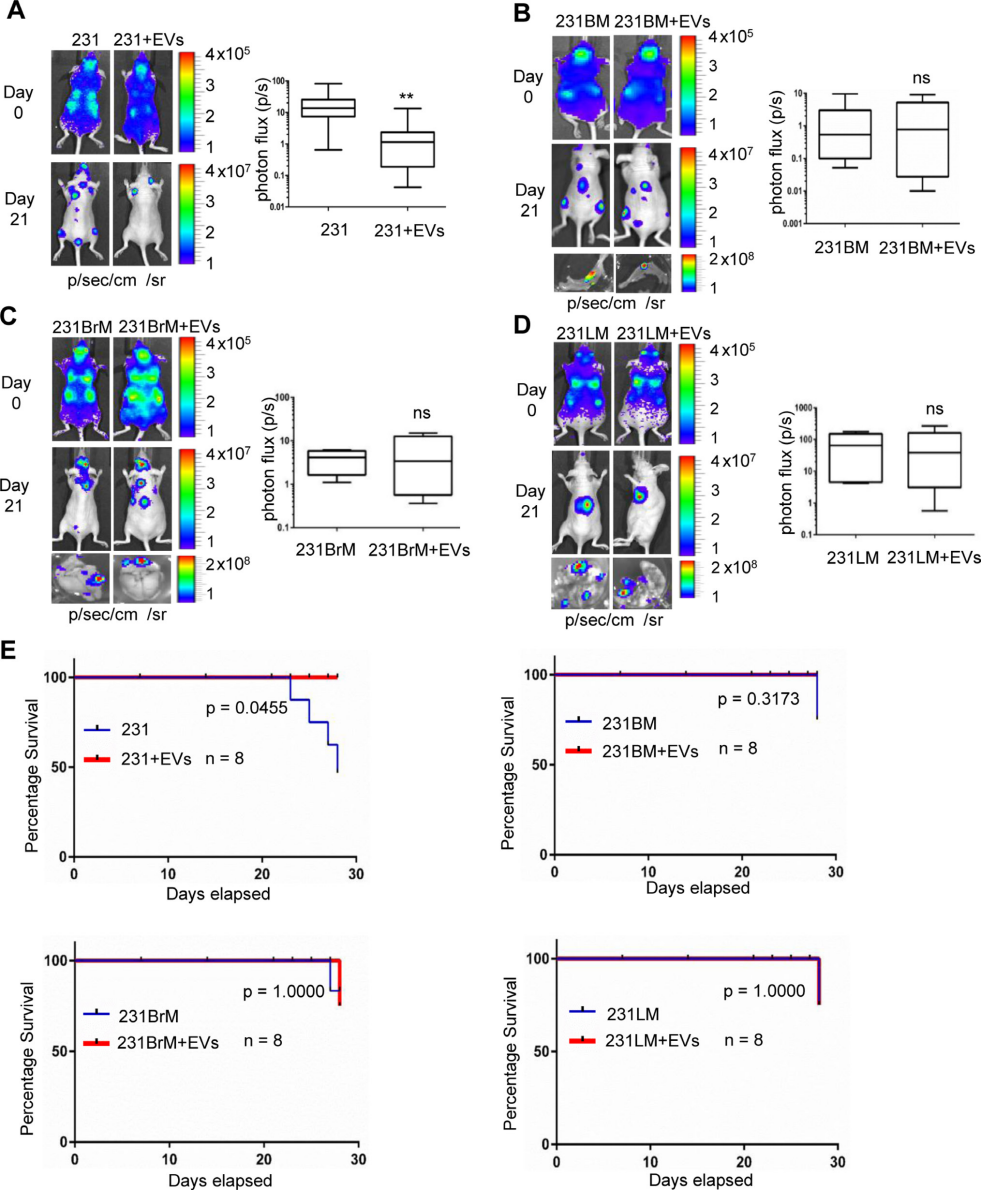
EVs suppress metastasis of MDA-MB-231 non-specific metastatic cells but not in organ-specific metastatic cells (**A**) left, bioluminescence imaging (BLI) images of the metastatic lesions of representative mice from each experimental group that received MDA-MB-231 (parental) and hMSC-EV-primed MDA-MB-231 cells respectively, *n* = 8. right, total photon flux of metastatic lesions was measured by BLI at the endpoint. (**B**, **C**, **D**) left, BLI images of the metastatic lesions of representative mice from each experimental group that received organ specific sublines (B) bone (231BM), (C) brain (231BrM) and (D) lungs (231LM) without or with hMSC-EV priming. right, total photon flux of metastatic lesions was measured by BLI at the endpoint. *n* = 8, ^**^, indicates *P* < 0.001. (**E**) Kaplan–Meier analysis for percentage survival of mice inoculated with parental and organotropic sublines versus hMSC-EV primed cells, *n* = 8.

Earlier studies from our group showed that extracellular vesicles derived from stressed human mesenchymal stem cells (hMSC-EVs) promoted primary breast tumor growth by transporting supportive miRNAs, growth factors and other cytokines [9, 13, 20, 21]. Towards, understanding the role of stromal cell secreted EVs on metastasis and dormancy, we examined the role of hMSC-EVs in metastasis of breast cancer *in vivo*. MDA-MB-231 cells (231 cells) that expressed luciferase were primed by incubating them with hMSC-EVs for 16 hrs. Cells were then transplanted into nude mice through intracardiac (IC) administration. Metastatic tumor growth was monitored by bioluminescence imaging every 7 days. As shown in Figure 1, successful IC injection was monitored by bioluminescence imaging of mice within 30 mins of injection (Day 0) which displayed distribution of cells all over the body (photon flux in the cranial region of Day 0 was used for normalization). After 3 weeks, metastatic tumor growth in various organs was prominent in mice that received 231 cells alone, whereas, mice receiving hMSC-EV-primed 231 cells showed 10 fold less bioluminescence (Figure 1A). *Ex-vivo* bio imaging of various organs revealed that mice that received hMSC-EV-primed cells displayed a reduced signal intensity in organs compared with un-treated cells (Supplementary Figure 1). Similar experiments to examine the effect of hMSCs-EVs on the organotropic sublines of MDA-MB-231 cells to bone (231BM), brain (231BrM) and lungs (231LM) by priming them with or without hMSCs-EVs were conducted. Interestingly, the metastatic potential of organotropic sublines was not affected by hMSCs-EV (Figure 1B–1D). *Ex-vivo* bioimaging of organs also did not show any significant difference in photon flux between treated and untreated mice (Figure 1B–1D, bottom part). Notably, the survival rate in mice receiving MDA-MB-231 cells alone was 50% by day 28, while 100% survival was observed in mice receiving MDA-MB-231 cells + hMSC-EVs. (Figure 1E). Hematoxylin and eosin (H & E) staining of the organs showed a higher grade of tumors in mice that received untreated cells compared with the hMSC-s-EV-primed cancer cells (Supplementary Figure 2). These results strongly indicate that hMSCs-derived EVs suppress the metastasis of parental MDA-MB-231 cells, and that hMSCs-EV co-injection decreases the mortality rate as well.

### hMSC-EVs exert similar primary tumor supportive effects on parental MDA-MB-231 breast cancer cells and their organotropic sub-lines *in vivo*

**Figure 2:**
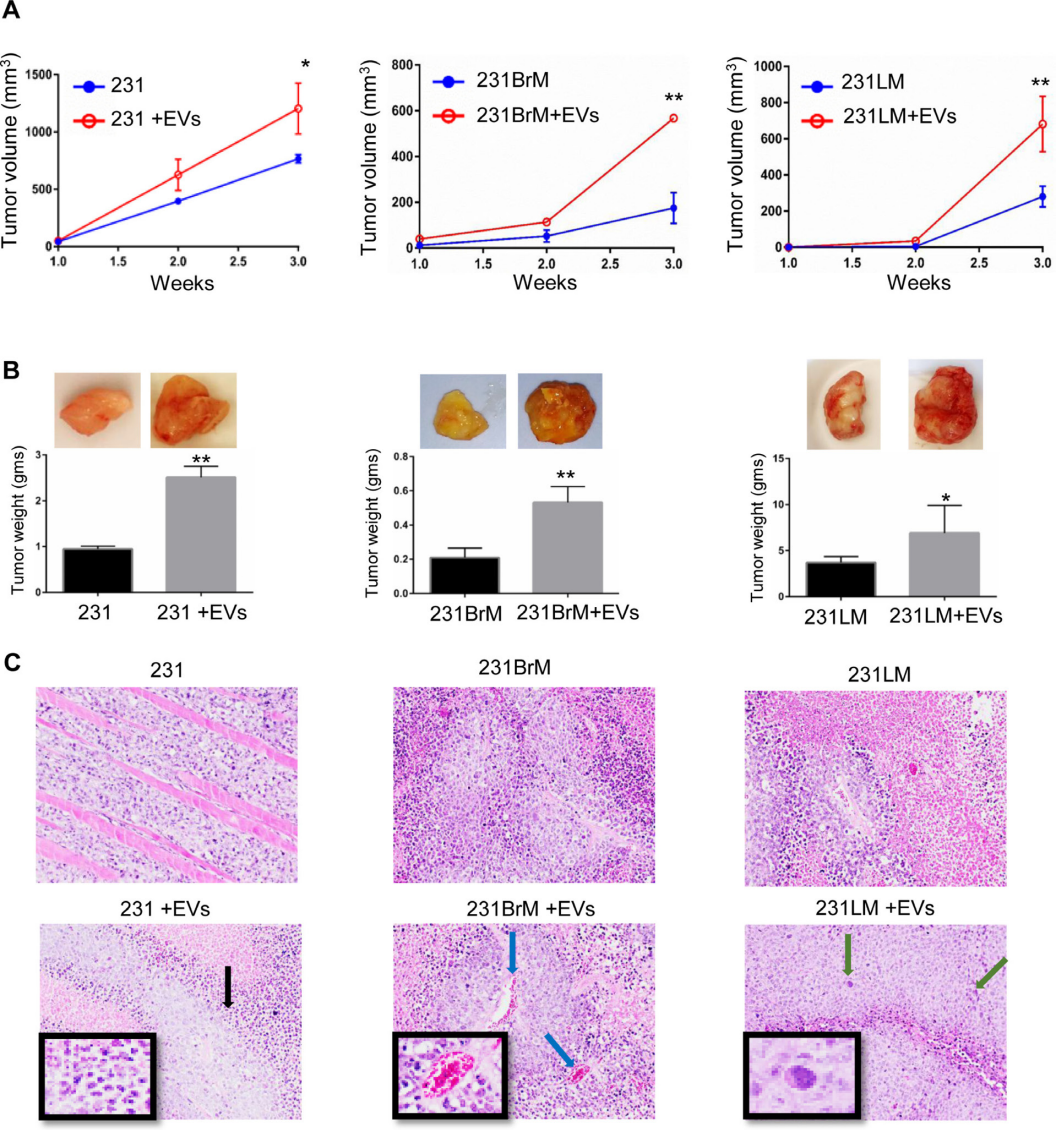
hMSC-EVs support primary breast tumor growth (**A**) tumor volume and (**B**) tumor weight, obtained from the NOD/SCID mice injected without or with hMSC-EV-primed MDA-MB-231 cells (left), organ-specific sublines to brain (middle), and lungs (right). Data are presented as the mean ± standard deviation. *n* = 6. (**C**) Representative images of hematoxylin and eosin staining of tumor sections showing more necrosis (black arrow), vessel formation (blue arrow), and pleomorphism (green arrow) in the hMSC-EV treated group. ^**^P < 0.001; ^*^P < 0.05.

To reproduce previous observations in orthotropic xenograft model [13], orthotropic xenograft assays were performed using untreated or hMSC-EV-primed 231 cells in NOD/SCID mice. Equal number of 231 cells (1 × 10^6^) were injected with matrigel into the mammary fat pads of two groups of mice. One group received cells alone, and other received cells primed with 50 μg of hMSC-EVs. Tumor growth in the EV-primed group was significantly faster than that in the group which received cells alone (Figure 2A), corroborating our previous observations. The mean tumor size in EV-primed group was 625 mm^3^ by 2 weeks after implantation, while in the untreated group it was 395 mm^3^ (Figure 2B). No metastasis was observed 6 weeks post-transplantation, and mice were sacrificed as required by the humane endpoints procedure in the IACUC-approved research protocol. Similar experiments to compare the trend in 231BM, 231BrM, and 231LM, that were primed with or without hMSCs-EVs were performed. Mice injected at mammary fat pads with EV-primed cells of all sublines formed tumors which were significantly larger in size and weight than those of mice injected with untreated cells (Figure 2A and 2B). Although H & E staining of the tumor sections did not show any significant morphological difference in tumors, the tumors from mice that received EV-primed cells had more necrosis, vessel formation, and pleomorphism (Figure 2C). These results confirm that effect of hMSCs-EVs on metastatic potential is not attributed.

### hMSC-EVs induce dormancy in parental but not in their organotropic sub-lines

**Figure 3:**
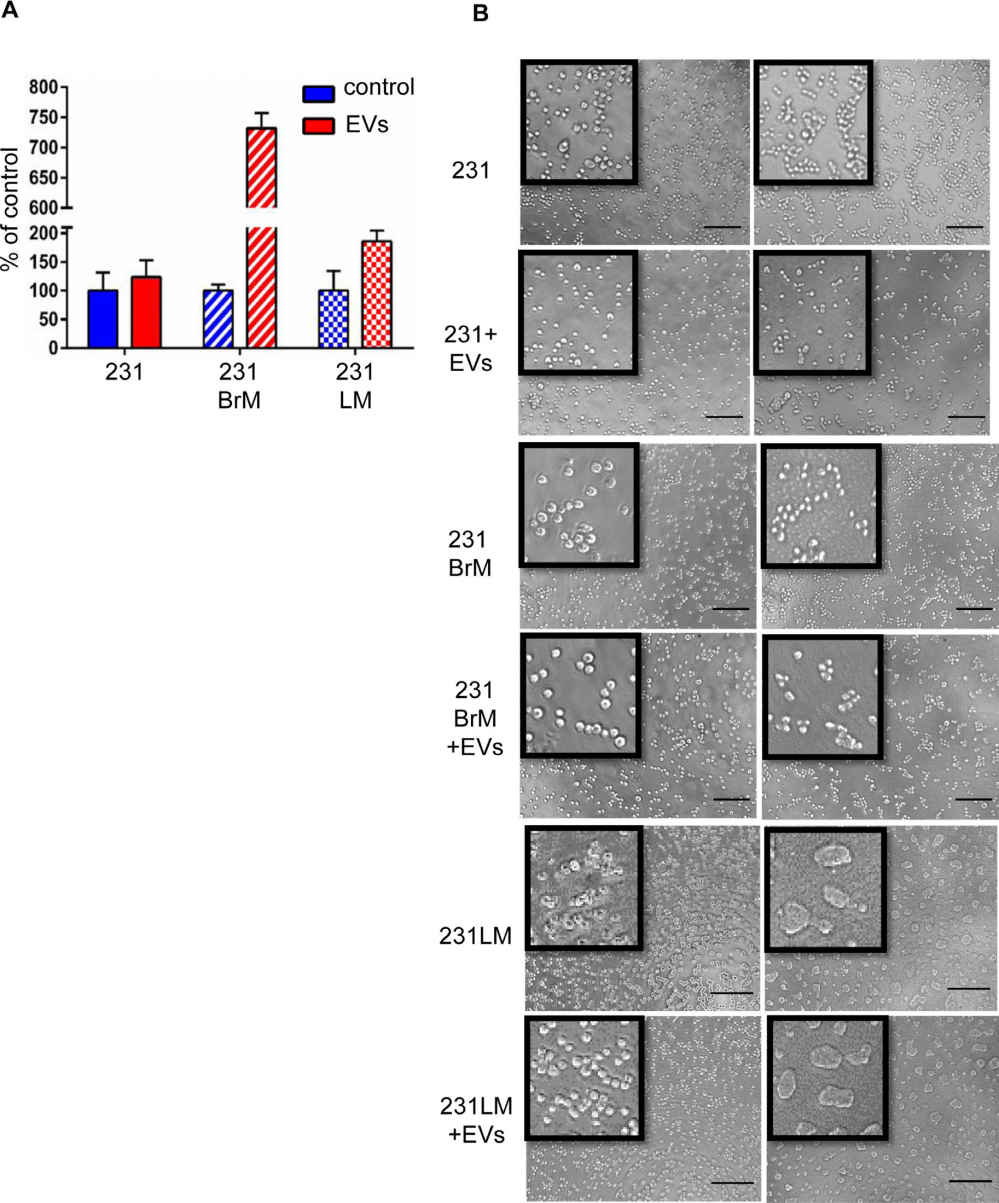
hMSC-EVs induces dormancy of parental but not in their organotropic sub-lines (**A**) Cyquant proliferation assay performed on parental cells and their organotropic sublines with or without hMSC-EVs. (**B**) 3D sphere-forming assay on parental cells and their organotropic sublines with or without hMSC-EVs.

To investigate if the metastasis suppression in parental cells is due to dormancy induced by hMSC-EVs, we performed proliferation and 3D culture assays. Earlier studies have shown that dormant tumor cells *in vivo* at metastatic sites remain isolated and quiescent, whereas highly metastatic cells form 3D spheres and proliferate in 3D *in vitro* assays [22]. A proliferation assay using DNA quantification demonstrated a significant increase in the proliferation of 231 cells primed with hMSC-EVs compared with 231BrM, and 231LM (Figure 3A). Furthermore, the 3D sphere formation assay showed that a high percentage of 231 cells primed with hMSC-EVs remain solitary when compared with unprimed 231 cells. However, there was no difference in sphere formation observed for primed/unprimed 231BrM or 231LM cells (Figure 3B). These results indicate that hMSC-EVs suppress the metastasis of parental MDA-MB-231 cells by inducing dormancy.

### miR-205 and miR-31 are up-regulated in breast cancer cells upon hMSC-EVs treatment

**Figure 4:**
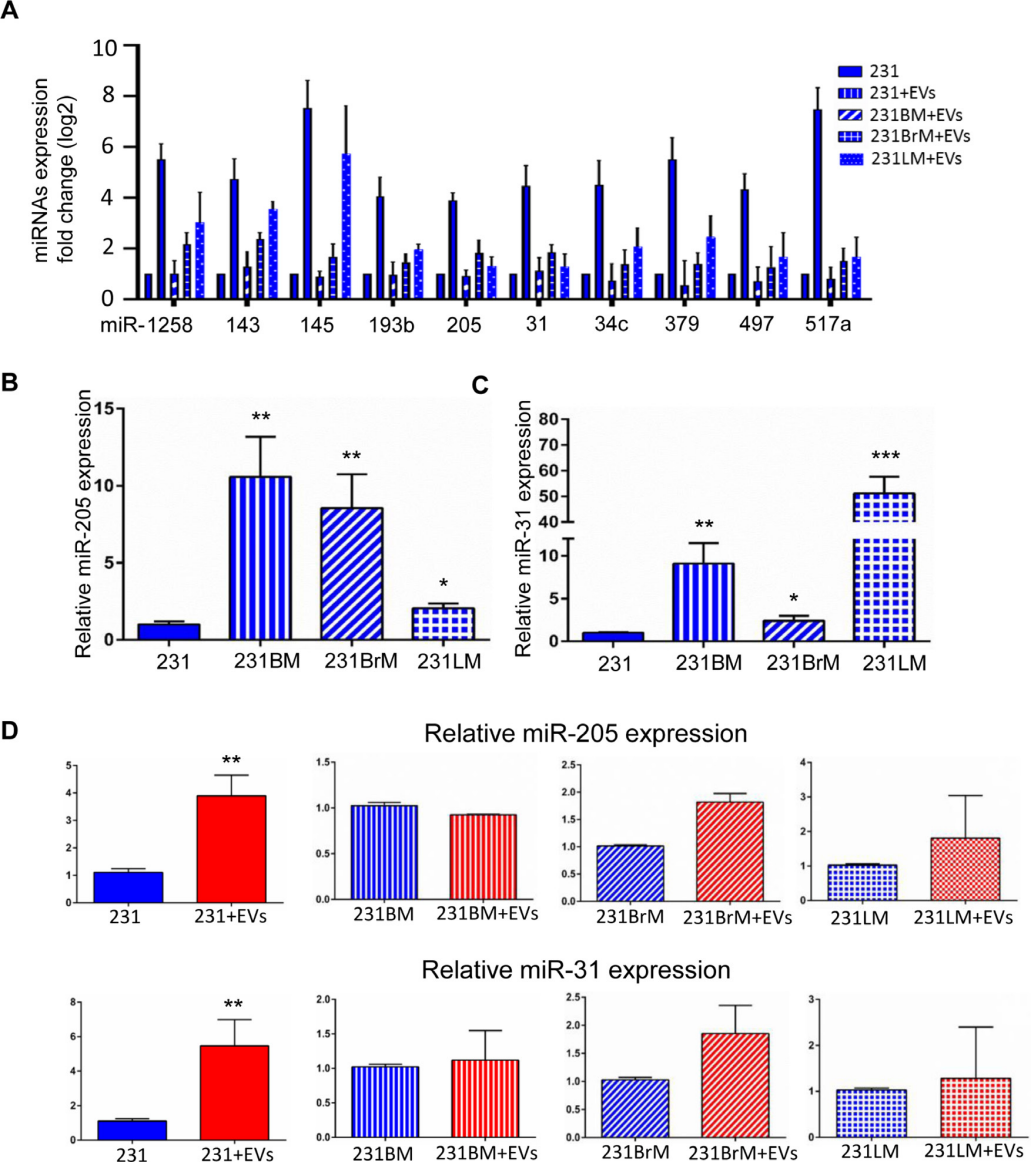
Identification of cargo/miRNA present in hMSC-EVs that affects dormancy phenotype (**A**) RNA expression of shortlisted 10 miRNAs in the parental and organotropic cell lines analyzed by RT-PCR. (**B**) The expression of miR-205 and (**C**) miR-31 were examined by qRT-PCR in MDA-MB-231, 231BM, 231BrM, and 231LM. (**D**) The expression of miR-205 (left) and miR-31 (right) were examined by qRT-PCR in MDA-MB-231, 231BM, 231BrM, and 231LM without or with treatment with hMSC-EVs. Data are represented as mean ± SEM, *n* = 3, ^***^P < 0.0001; ^**^P < 0.001; ^*^P < 0.05.

Data from xenograft assays of primary and metastatic tumor growth prompted us to identify the factors in EVs that differentially regulate metastatic potential. As shown previously by our lab and others, the regulatory cargo of EVs consists of miRNAs, metabolites, proteins and DNA [21, 23, 24]. Given that all the cell lines are from the same genetic background and that the observations of the effect on metastasis is rather specific, we speculated that the cargo has a role in epigenetic regulation. While all or any of the macromolecules in the EVs could play a role in the effect seen with metastastic cells, we chose to focus on miRNAs for their role in epigenetic regulation. To identify specific miRNAs that would have an effect on metastasis, a sequential analysis of next-generation sequencing data obtained from hMSC-EVs (as described in [13]) was performed. All highly expressed miRNAs were subjected to a manual Pubmed search to identify the groups of miRNA that had demonstrated roles in metastasis. This analysis narrowed our list from 157 miRNA to 36 miRNAs with known roles in metastasis (Supplementary Figure 3). Next, we made a qPCR panel with these 36 miRNAs and assayed for their expression in all 4 cell lines of interest in this study. Comparing RNA isolated from 231, 231-BM, 231-BrM, and 231-LM cells that were treated with or without EVs against the shortlisted miRNA panel revealed that there were 10 miRNAs whose expression was significantly altered upon hMSC-EVs treatment in 231 cells (Figure 4A). Of these 10 miRNAs, miR-205 and miR-31 were chosen for further study because the MDA-MB-231 parental cell line expressed significantly lower level of these miRNAs compared with the 231 sublines (Figure 4B and 4C). We found that treatment with hMSCs-EVs resulted in increased miR-205 and miR-31 expression in the parental line compared with the sublines (Figure 4D). These results suggest that miRs-205 and 31 may contribute to the metastatic ability of 231 cells when treated with EVs.

### UBE2N/Ubc13 is the downstream target of miR-205 and miR-31 *in silico*

**Figure 5:**
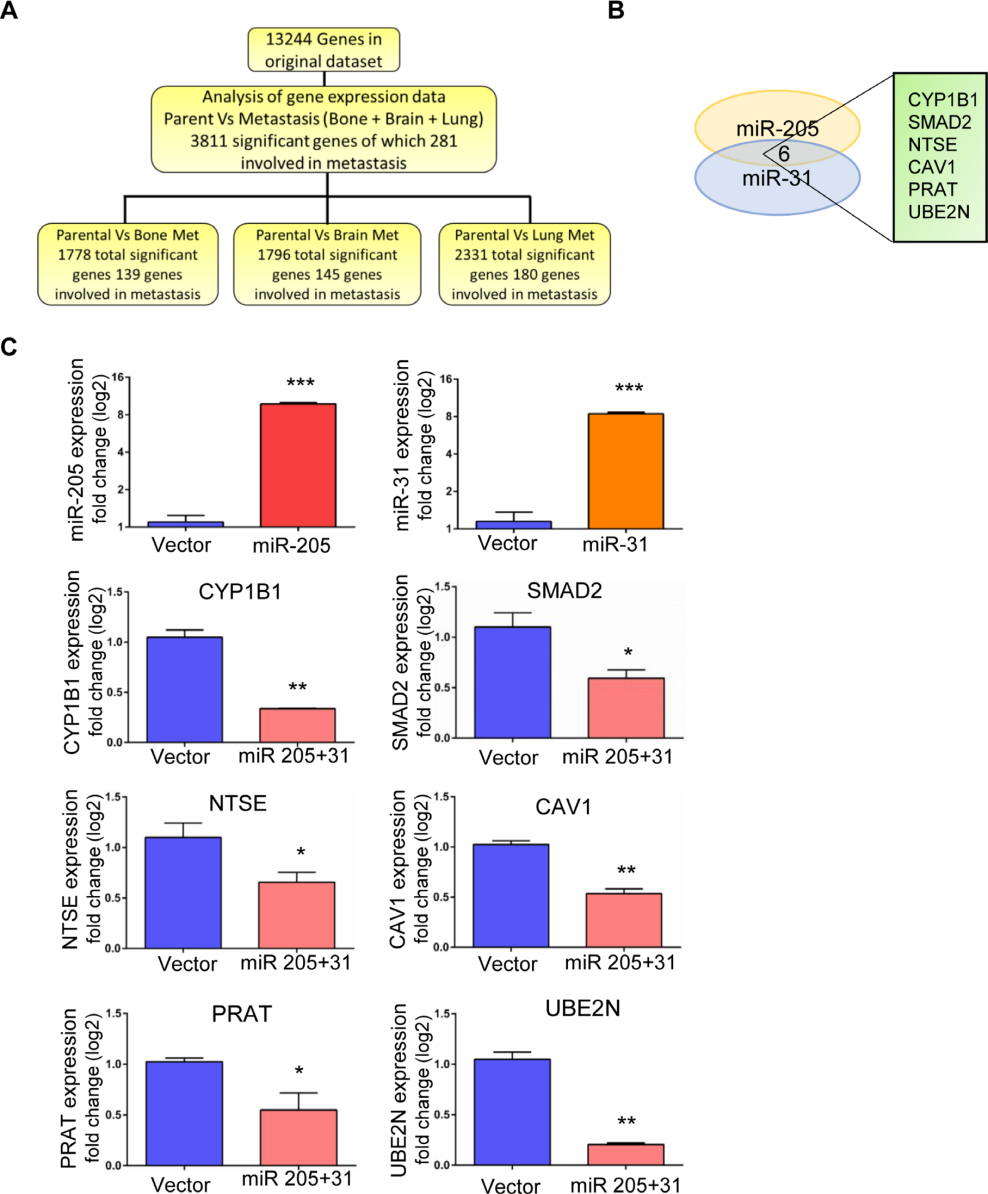
Identification of downstream target for miR-205 and miR-31 *in silico* and *in vitro* validation (**A**) Schematic of data analysis (**B**) Venn diagram showing the common target genes for miRNAs 205 and 31. (**C**) Real-time PCR data showing the over expression of miR-205 and miR-31 by lentiviral transductions and the significant down-regulation of the target genes upon co-expression of miRs 205 and 31.

**Figure 6:**
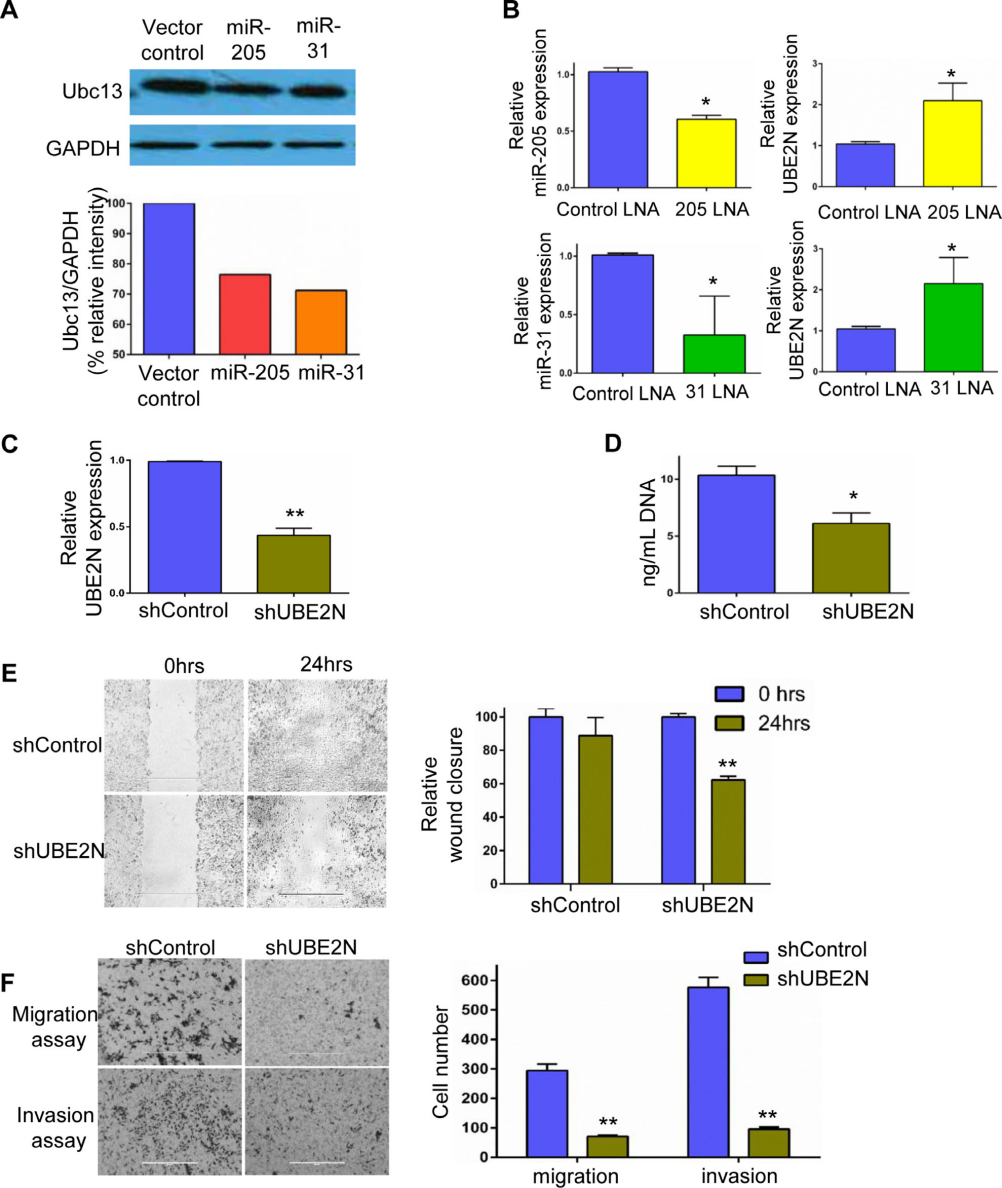
Identification of downstream target for miR-205 and miR-31 *in silico* and *in vitro* validation (**A**) Western blot analysis of UBE2N/Ubc13 in MDA-MB-231 cells upon over-expression of miRNAs-205 and 31. (**B**) MDA-MB-231 cells were transfected with miR-205 and miR-31 locked nucleic acids, and the successful silencing is shown in the left panels, while the corresponding expression levels of UBE2N/Ubc13 as measured by RT-PCR is shown in the right panels. (**C**) Relative expression of UBE2N/Ubc13 in silencing control and shUBE2N, as detected by real-time PCR. (**D**) Cyquant proliferation assay revealed reduced cell growth in UBE2N/Ubc13-silenced clones compared with vector control. (**E**) Representative images of wound scratch assay and corresponding quantification showing migration of MDA-MB-231 cells in vector control and UBE2N/Ubc13-silenced cells. (**F**) *In vitro* cell invasion assay in matrigel chambers. The left panel has representative fields of invaded cells, while the corresponding quantification is shown in the right panel. Data are represented as mean ± SEM, *n* = 3, ^***^P < 0.0001; ^**^P < 0.001; ^*^P < 0.05.

To understand the regulatory role of miR-205 and miR-31 in breast cancer metastasis, we studied their common downstream targets and further investigated the underlying molecular mechanisms as metastatic-suppressive miRNAs. Target genes were identified by analysis of clinical microarray cohort data (GEO database- GSE12237, GSE2603). Out of 13,244 genes in the original dataset, we identified 3,811 significantly differentially expressed genes between 231 cells and their highly metastatic variants to bone, brain, and lung, of which 281 were involved in metastasis. A schematic for data analysis is shown in Figure 5A. Twenty-seven genes were selected based on differential expression in all three metastatic variants (Supplementary Figure 4). To further narrow down the list of target genes, we used *in silico* algorithms (TargetScan, PicTar, miRanda) to predict common target genes. Six of the genes were common targets of miR-205 and miR-31 (Figure 5B). Comparison of our *in silico* data mining output to previously published studies revealed an association between low miRNA 205 levels and worse relapse-free survival rates [25]. Additionally, miR-31 expression has been linked to the metastatic potential in cancers, such as prostate, gastric, and colon cancers [26–28]. To examine the effect of miR-205 and 31 on the expression of common targets CYP1B1, SMAD2, NTSE, CAV1, PRAT, UBE2N/Ubc13, we ectopically expressed these miRNAs in 231 cells by transient transfection followed by western blot analysis. As shown in Figures 5C, 6A and Supplementary Figure 5, we found that both miR-205 and miR-31 significantly suppressed the expression of all the target genes in these cells. Among the common target genes, we focused on highly regulated UBE2N/Ubc13 gene, which is known to have a critical role in metastasis [29] and was previously identified as a target of miR-205 [25].

### miR-205 and miR-31 suppress the expression of UBE2N/Ubc13 gene

Next, qRT-PCR to analyze the endogenous mRNA levels of UBE2N/Ubc13 with and without treatment with EVs in all the breast cancer cell lines was performed. Using TargetScan and other bioinformatics tools, we found potential miR-205 and miR-31 binding sites on UBE2N 3’UTR sequence as shown in schematic (Supplementary Figure 6A). Endogenous levels of UBE2N/Ubc13 in 231 cells were significantly low when compared with their sublines. Upon EV treatment there is a significant down-regulation of UBE2N/Ubc13 that is observed only in 231 parental cells (Supplementary Figure 6B). To investigate if UBE2N/Ubc13 is regulated by miR-205 and miR-31, we ectopically expressed the miRNAs by lentiviral transfection in 231 cells. As shown in Figures 5C and 6A, ectopic expression of miRNAs 205 and 31 significantly suppressed the expression of UBE2N/Ubc13. On the other hand, transfection of miR-205 and miR-31 locked nucleic acids (LNAs) significantly enhanced UBE2N/Ubc13 expression in 231 cells (Figure 6B). These results strongly support that miR-205 and miR-31 suppress the expression of the UBE2N/Ubc13 gene.

### UBE2N/Ubc13 downregulation suppresses the proliferation, migration, and invasion of breast cancer cells *in vitro*

To test the role of UBE2N/Ubc13 in tumor metastasis, cell lines possessing stable knockdown of UBE2N/Ubc13 were generated using shRNA construct. Confirming knockdown, qPCR expression assays demonstrated a 60% downregulation in UBE2N/Ubc13 expression in shUBE2N-transfected cells compared with those receiving control vector (Figure 6C). Furthermore, proliferation assays using DNA quantification demonstrated a 30% lower proliferation in cells with UBE2N/Ubc13 knockdown (shUBE2N) compared with control vector cells (Figure 6D). Since breast cancer metastasis is directly associated with the motility of the cells, the effect of UBE2N/Ubc13 silencing on wound healing and migration was investigated. As shown in Figure 6E, a time course analysis of wound closure showed that the cell layer was re-established significantly faster in the control vector group than the shUBE2N group. After the cells were incubated for 18 hrs in the transwell assay system, the number of control vector cells that had moved through the membrane of the chamber was 4.2-fold higher than the number of shUBE2N cells (Figure 6F). Similarly, shUBE2N cells were observed to be less invasive. After the cells were incubated for 24 hrs in the transwell assay system with matrigel inserts, the number of control vector cells that invaded through the membrane of the matrigel chamber was 6.1-fold higher than that of shUBE2N cells (Figure 6F). These results suggest that UBE2N/Ubc13 induces a strong pro-migratory effects on cells, and high expression of it in organotropic sublines explains the metastatic capability of cells. Our results also strongly suggest that miR-205 and 31 transported through hMSC-EVs downregulate UBE2N/Ubc13, thereby suppressing the metastatic ability of 231 cells.

### UBE2N/Ubc13 upregulation correlates with high risk/poor prognosis breast cancer

**Figure 7:**
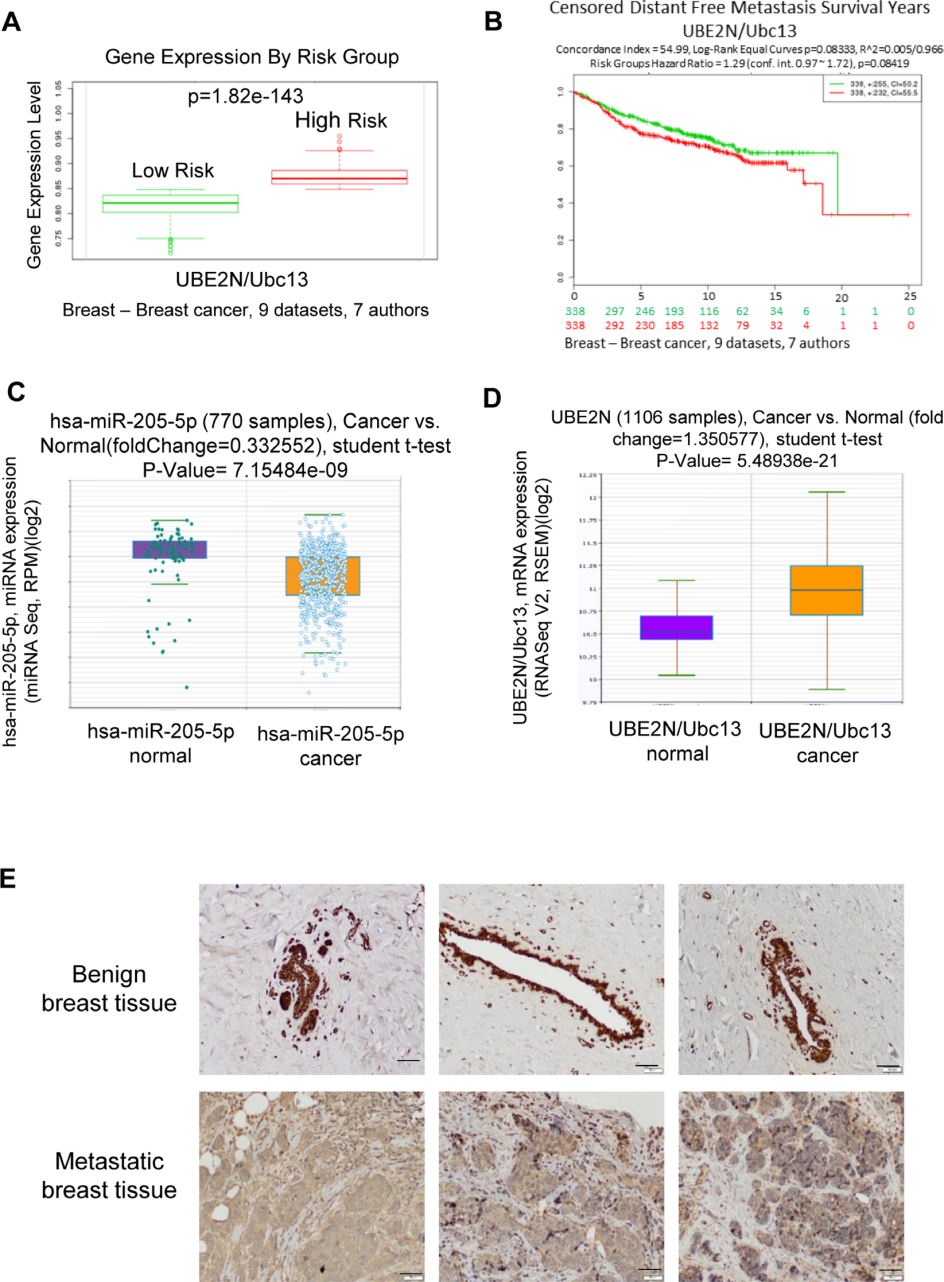
UBE2N gene expression levels from human breast cancer samples (**A**) UBE2N gene expression levels from human breast cancer samples from 9 datasets, *p* = 1.82e-143 (**B**) UBE2N expression and corresponding distant free metastasis survival years of human breast cancer patients from 9 datasets, *p* = 0.08419 (**C**) miR-205 expression in 770 samples of normal vs breast cancer, *p* = 7.15484e-09 (**D**) UBE2N expression in 1106 samples of normal vs breast cancer, *p* = 5.48938e-21 (**E**) Benign and metastatic breast biopsy samples were obtained from three different human patients. Immunohistochemistry was performed on specimens using anti-Ubc13 or an IgG control antibody. Positive cells were visualized by DAB staining. Scale bar, 50 μm.

A review of previously published data on UBE2N/Ubc13 expression via tissue microarray was performed. Microarray profiling of microRNAs in breast cancer was completed using Starbase v2.0 (http://starbase.sysu.edu.cn/) [30, 31]. Utilizing the same datasets, the gene expression of UBE2N/Ubc13 was found to be significantly increased in high-risk (early disease-related death) breast cancer patients (Figure 7A and 7B), which correlated with a lower expression of miR-205 (Figure 7C) and a higher expression of UBE2N/Ubc13 mRNA expression (Figure 7D). Although UBE2N/Ubc13 expression level alone is not an independent predictor of survival (Figure 7B), these data from patient samples are in agreement with *in vitro* results assessing the proliferation, migration, and invasion potential of MDA-MB-231 cells possessing downregulated UBE2N/Ubc13 expression, and overall substantiate the relevance of UBE2N/Ubc13 in breast cancer aggressiveness.

### Immunohistochemical (IHC) staining for Ubc13 in human breast cancer tissues

IHC analysis was performed to investigate the expression of Ubc13 in paraffin-embedded human mammary tissue from benign breast removed for macromastia and from metastatic breast cancer patients using blocks obtained from institutional biorepository. The Ubc13 staining is shown in Figure 7E. The luminal epithelial cells from benign breast tissue show diffuse cytoplasmic and nuclear staining, which is in contrast to the background stromal fibroblasts, which express nuclear positivity but lack the cytoplasmic staining. In sections from the metastatic breast tumors, there is diffuse nuclear and cytoplasmic positivity for Ubc13, which again contrasts from the background benign stromal cells, which show only nuclear positivity (Figure 7E).

## DISCUSSION

Previously published studies demonstrated that stressed hMSC-EVs act as carriers that transport miRNAs and support primary breast tumors recaptilating the nutrient deprived core [9, 20, 21, 23, 32, 33]. However, a role of EVs in the dissemination and colonization of breast cancer cells in the metastatic niche has not been well characterized. Since metastatic lesions in breast cancer patients often arise in bone, lung, liver, and brain, the first step was to determine the effect of EVs on such organ-committed BC cells compared with the non-committed, but metastatic, parental cell line. This report demonstrates that hMSC-EVs regulate breast tumor metastasis in non-organ committed metastatic cells when compared with organotropic metastatic cells. Differences between the effects of hMSC-EVs on parental 231 cells and organ-committed cells are quite characteristic of experiments involving stem cells, with reasons suggested for the discrepancies ranging from toll-like receptor expression patterns to the timing of the administration of MSCs [34, 35].

The cargo of EVs can consist of several disparate species of macromolecules. Of particular interest are microRNAs, which have the ability to regulate genes involved in multiple cellular processes, including metastasis. Several stages of bioinformatics analysis to identify unique miRNA that may explain the differential effect of EVs on parental 231 cells, identified several miRNAs of interest (Supplementary Figure 3A and 3B); two miRNAs, miR-31 and miR-205, were specifically associated with breast cancer metastasis [36–38]. As shown in Figure 4A, both miR-205 and miR-31 were downregulated in 231 cells compared with the cell lines derived from organ metastases. Expectedly treatment of 231 cells with hMSC-EVs, followed by RT-PCR analysis of microRNA expression showed a significant increase in miR-31 and miR-205 expression (Figure 4C), confirming that the observed effects of hMSC-EV exposure can be attributed, at least in part, to miRNA expression, as previously demonstrated by our lab and others.

Considering the ability of a single miRNA to suppress a large number of target genes, next logical step was to identify the targets for miRNAs 31 and 205 that would have an effect on metastasis. Bioinformatic analysis to identify targets with 3’UTRs of both miR-205 and miR-31 regulatory sequences and that were also overexpressed in organ-specific 231 variants (Figure 4B) was performed. Several targets were identified of which initial screening assays have led us to focus on UBE2N/Ubc13 as described in results section. UBE2N/Ubc13, which catalyzes the polyubiquitination of target proteins, has been previously identified as a potential oncogene and is associated with tumorigenesis through its interaction with the *c-FOS* promoter and NF-κB [39, 40]. In the lung, UBE2N/Ubc13 has previously been shown to control both metastasis and colonization [41]. Additionally, meta-analysis of UBE2N/Ubc13 gene expression demonstrated an association between high risk breast cancer phenotype (Figure 7A). Furthermore, decreased expression of UBE2N/Ubc13 upon overexpression of miR-205 or miR-31 was consistent with a previous study that demonstrated that miR-205 overexpression silenced UBE2N/Ubc13 [29]. If relative levels of miR-205 and miR-31 are significantly higher in sublines compared to parental cells (2 to 50 fold, Figure 4B–4C), levels of Ubc13 protein expression are relatively stable between parental and sublines (2 to 4 fold, Supplementary Figure 6B). This can be explained by the ability of cells to regulate and stabilize the expression of Ubc13 over time and induce a selection between clones. It can also be speculated that cells are using other mechanisms of regulation to maintain a functional level of Ubc13.

Our observation that hMSC-EVs suppress the metastasis of parental 231 cells yet do not significantly affect the metastasis of organ-specific 231 cells strongly suggests a connection to the epithelial-mesenchymal transition (EMT). miR-205, in particular, has been associated with EMT and the regulation of Notch 2 signaling [42]. Correspondingly, STAT3 transcriptional activity is required to repress UBE2N/Ubc13 expression, whereby it promotes pre-metastatic niche formation [43]. The effect of UBE2N/Ubc13 downregulation on the invasion and migration observations are supportive of the seed and soil hypothesis first introduced by Stephen Paget [44]. Despite evidence for sites that are permissive to tumor cell engraftment in advance of any exposure to tumor-derived factors, for example leukemic cells homing to sites expressing stromal-derived factor-1 [45], our results are more in line with the hypothesis that cancer and stromal cells co-evolve for metastasis. The establishment of metastases requires not only cancer cell extravasation into the distant tissue, but also the remodeling of the extracellular matrix, recruitment and co-opting of stromal cells to support tumor growth, and immune system evasion.

Based on our observations that UBE2N/Ubc13 is overexpressed in organotropic cell lines and previous published results, it appears as if overexpression of miR-31 and miR-205 may act as regulators of initial local invasion, where decreased expression of UBE2N/Ubc13 and the subsequently suppressed activation of the downstream effector NF-κB is unable to suppress apoptosis or increase MMP1 expression [40, 41], leading to overall decreased metastasis of MDA-MB-231 cells. However, the effect of miR-31 and miR-205 on organotropic cell lines is moderated by their higher expression of UBE2N/Ubc13. While it has been known for many years that factors produced by primary tumors could lead to premetastatic niche formation, no consensus has been reached on which signaling pathways should be prioritized for the development of new cancer therapeutics. Therefore, studies of this nature are highly relevant, as they could provide us with a better understanding of the differences between the pre-existing metastatic niche and the induced (pre-metastatic) niche [43].

## MATERIALS AND METHODS

### Cells and cell culture

MDA-MB-231 (231) and organotropic sub-lines (231BrM-2a, 231LM-4175, 231BM-1833 from Dr. Massague) were provided by Dr. Kounosuke Watabe. Cells were labeled with firefly luciferase through lentiviral transduction and the clones were selected for *in vivo* experiments based on photon flux (>10^9^) of bioluminescence image. All the cells were cultured in Dulbecco's modified Eagle's medium (DMEM) supplemented with 10% FBS, 1% antibiotics (penicillin/streptomycin).

### Extracellular vesicles (EVs) isolation and characterization

EVs were isolated and characterized as mentioned in our earlier publication [13]. Briefly, hMSCs were cultured in serum containing media in two-layered cell factories. Serum media was replaced with serum deprived media and cells were grown for period up to 45 days. Conditioned media of centrifuged to eliminate cell debris. The supernatant was concentrated to a final volume of 5 ml and ultra-centrifuged at 15,000 g for 1 h at 4°C to remove large vesicles, the supernatant which contain the EV fraction was further subjected to ultracentrifugation at 110K g overnight at 4°C. EV pellets were washed with phosphate buffered saline and ultracentrifuged at 110K g overnight at 4°C and pellets were resuspended in 100 μl of PBS and aliquots were stored at –80°C. EVs, were characterized using NanoSight LM10 system (NanoSight Ltd, Amesbury, UK).

### Animal experiments

Female NOD/SCID mice, and female *NU/NU* mice, 6–7 weeks old were purchased from Charles River Laboratories (Wilmington, MA). All animal studies were conducted in accordance with NIH animal use guidelines and a protocol approved by UMMC animal care committee. For orthotopic model, NOD/SCID mice were divided randomly into 8 groups (*n* = 4). 231, 231BM, 231BrM, 231LM cells were primed with or without serum deprived hMSCs-EVs (50 μg/10^6^ cells) for 16 hrs. Cells were trypsinized, washed twice with phosphate buffered saline (PBS) and suspended in 100 μl matrigel and were injected into right and left mammary fat pads of each mice (*n* = 8). Tumor size was measured every week using calipers and the volume of the tumor was calculated using the formula *V* = (4/3) πa^2^b, where ‘a’ is the shorter radius in mm and ‘b’ is longer radius in mm. Mice were monitored for metastasis every week starting from 3 weeks of injection using bioluminescence (BLI) imaging by IVIS Xenogen bioimager (Caliper). For metastatic model, *NU/NU* mice were divided randomly into 8 groups (*n* = 8). All the cells were primed with or without EVs as mentioned above. 50,000 cells suspended in 80 μl to 100 μl PBS were injected into the left cardiac ventricle. To confirm the successful injection, the mice were injected with luciferin through i.p. and subjected to BLI imaging in IVIS within 30 mins of i.c. injection. The photon flux was measured every week to monitor the metastasis progression. At the end point of this study, mice were sacrificed and necropsy was performed and organs were collected and incubated in 0.5 mg/mL luciferin for 10 to 15 mins and photon flux was measured. Part of the organs were fixed in PFA for histochemistry studies.

### Plasmids

The plasmids expressing hsa-miR-205 and hsa-miR-31 precursors in lentiviral pCDH-CMV-MCS-EF1-copGFP vector (System Bioscience) were provided by Dr. Yin-Yuan Mo (University of Mississippi Medical Center). The lentiviral constructs with puromycin for shRNA plasmid-A and shRNA plasmid UBE2N were purchased from Santa Cruz Biotechnology, Inc. (Texas, USA).

### Transfection

For over expression of miRNAs 205 and 31, cells were transfected with Lipofectamine 2000. For down regulation of miRNA, cells were transfected with Locked nucleic acids (LNAs) (Exiqon, Woburn, MA) using Lipofectamine 2000 according to manufacturers protocols. LNAs were used at a concentration of 20 pmol/well in a 12 well plate. miRCURY LNA inhibitors sequences were: hsa-miR-205-5p 5′-3′ AGACTCCGGTGGAATGAA/36-FAM/; hsa-miR-31-5p 5′-3′ GCTATGCCAGCATCTTGCC/36-FAM/. For UBE2N silencing, shControl and shUBE2N were transfected into cells with Lipofectamine 2000 and after 48 hrs cells were selected using 2 μg/mL Puromycin for 2 weeks.

### Bioinformatic analysis

We performed a sequential analysis of next-generation sequencing data obtained from hMSC-EVs (as described in [13]). Briefly, The EVs small RNAs were processed to generate a cDNA library, which was then used for deep sequencing. The mappable miRNAs from human miRBase were sorted based on the copy number, function and *z* score. 157 highly expressed miRNAs were selected and subjected to a manual Pubmed search to identify their roles in metastasis. This literature-based search narrowed the selection to 36 miRNAs which were further subjected to a qPCR validation (see schematic in Supplementary Figure 3).

Microarray profiling of microRNAs in breast cancer was completed using Starbase v2.0 (http://starbase.sysu.edu.cn/) and its Pan-Cancer Analysis Platform to decipher Pan-Cancer Analysis Networks of miRNA-31 and 205 in association of UBE2N gene expression profiles of 9 breast cancer datasets from The Cancer Genome Atlas (TCGA) Data Portal.

### Real-Time PCR

For miRNAs studies, total RNA was extracted from cells using miRVana kit (Ambion, Grand Island, NY) according to manufacturer's protocol. Total RNA (500ng) was reverse transcribed to cDNA using miScript II RT kit (Qiagen, Valencia, CA). Real-Time PCR was performed using miScript SYBR Green PCR kit in CFX96 Real-Time PCR system. For UBE2N studies, total RNA was reverse transcribed to cDNA using iScript cDNA synthesis kit (BioRad, CA) and real-time PCR was performed using SYBR Green PCR master mix (Thermo Fischer, MA). The following primes were used to amplify the miRNAs and genes – hsa-miR-205 5′- TCCTTCATTCCACCGGAGTCTG-3′; hsa-miR-31 5′- AGGCAAGATGCTGGCATAGCT-3′;

UBE2N Fwd- CCAGAAGAATACCCAATGGCAG; UBE2N Rev- GCTGGGGACCACTTATCTTTCA

### Wound scratch/migration assay

Wound healing assay was performed to study the cell motility. Cells were seeded in a 12 well plate and allowed to form a confluent monolayer. The cell layer was then scratched vertically with a 20 μL pipette tip. The cell debris was removed from the wells by washing three times with phosphate buffered saline (PBS). DMEM media supplemented with antibiotics (penicillin/streptomycin) and 10% FBS was added. Each well was divided into 6 parts and images of wound closure were capture at 0, 2, 6, and 24 hrs. All the images were analyzed using imageJ software calculating the area of the open wound at different time points.

### Transwell migration and Invasion assays

Cell motility was measured using 8 μm ThinCert cell culture insert (Greiner Bio, Germany). Cells were seeded (25,000 cells per insert) in serum free media. 10% DMEM was added to the lower chamber. An invasion assay was performed following the same procedure, with an exception that the ThinCert was coated with 25 μg matrigel (BD Biosciences, CA, USA). The plates were incubated in cell culture incubator for 10 hrs and 18 hrs for transwell and invasion assays respectively. After the time point, cells on the top layer of ThinCert were removed by wiping with a cotton swab. Cells at the lower side of the membrane were fixed with 4% PFA for 10 mins and stained with 0.1% crystal violet for 1hr rinsed three times with PBS and images were captured using microscope. Values were obtained by counting six fields per membrane and represented the average of two independent experiments.

### Western blotting

Cells were harvested and protein lysate was collected as previously described [46]. The protein concentration was determined by Bicinchoninic acid assay kit (Thermo Scientific, Rochester, USA) and 25 μg proteins were separated through SDS-PAGE using 4–12% Bis-Tris gel. The separated proteins were transfered onto PVDF membrane (GenHunter Corp, Nashville, USA). The membrane was blocked with 5% non-fat milk and probed with UBE2N/Ubc13 primary Ab (1:1000, R&D Systems, UK). The membrane was incubated with secondary Ab conjugated with HRP, incubated with ECL western blotting substrate and exposed on CL-Xposure films (Thermo Scientific, Rochester, New York). Films were revealed using a Kodak M35-A X-OMAT processor.

### Hematoxylin and Eosin staining and immunohistochemistry

Breast tumors and organs from different groups were excised and fixed in 10% PFA and in 70% ethanol. Tissue paraffin embedding, sectioning and H & E staining were performed by the Histology Core facility, Department of Pathology, UMMC. The stained slides were evaluated by pathologist (KVA) who was blinded to the treatment. For immunohistochemistry, human benign and breast biopsy specimens were obtained from pathology repository. Paraffin-embedded specimens were deparaffinized in xylene, subjected to heat-mediated antigen-retrieval in 10 mM sodium citrate (pH 6.0), permeabilized in 0.2% Triton X-100 (Sigma) and blocked in 5% donkey sera. Ubc13 was detected using anti-Ubc13 antibody (Thermofischer) (1:100) and an HRP-conjugated donkey anti-rabbit secondary (1:250, Abcam), amplified with AB reagent (Vectastain) and detected using DAB reagent (Vector Laboratories). Images were acquired using a Nikon Eclipse 80i microscope.

### Proliferation assay

Cells were seeded at a density of 5,000 cells/well in a 96-well plate. DMEM without serum was used and the cells were allowed to proliferate up to 24 hrs. At the end of time point, cells were washed with PBS and the plate was frozen for 20 minutes. Cells were treated with the CYQUANT cell Proliferation assay reagent (Life Technologies) according to the manufacturer's instructions. Fluorescence was measured using Biotek Synergy 4 plate reader (excitation 480 nm, emission 530 nm) (Winooski, VT) and expressed as percentage relative to control. Each experiment was performed with eight replicates, and the experiments were repeated two times.

### Cell proliferation assay in 3D system to study tumor cell dormancy

MDA-MB-231 (231) and organotropic sub-lines (231BrM-2a, 231LM-4175, 231BM-1833) were seeded at a density of 10,000 cells/well in a 96 well plate coated with ELP-PEI as described in [47, 48]. The cells were pre-treated with or without EVs. Images were captured using digital camera. Real time proliferation and sphere formation is tracked for 24 hrs at 6 different points per well by time-lapse imaging and measured using ImageJ digital analysis software.

### Statistical analysis

The data are represented as mean ± SD of the samples. All experiments were performed three times. Animal experiments were performed with 8 mice per group. Statistical analysis was performed using non-paired *t*-test, and *p* value < 0.05 was considered statistically significant.

## SUPPLEMENTARY MATERIALS FIGURES


