# Supplementary material for: Stromal cell extracellular vesicular cargo mediated regulation of breast cancer cell metastasis via ubiquitin conjugating enzyme E2 N pathway
Source: Oncotarget. 2017 Nov 10;8(66):109861–76. doi: 10.18632/oncotarget.22371 (PMC5746349; doi:10.18632/oncotarget.22371)
Supplement: Supplementary file 1 [file oncotarget-08-109861-s001.pdf]

Stromal cell extracellular vesicular cargo mediated regulation of breast cancer cell metastasis via Ubiquitin Conjugating Enzyme E2 N pathway

SUPPLEMENTARY MATERIALS

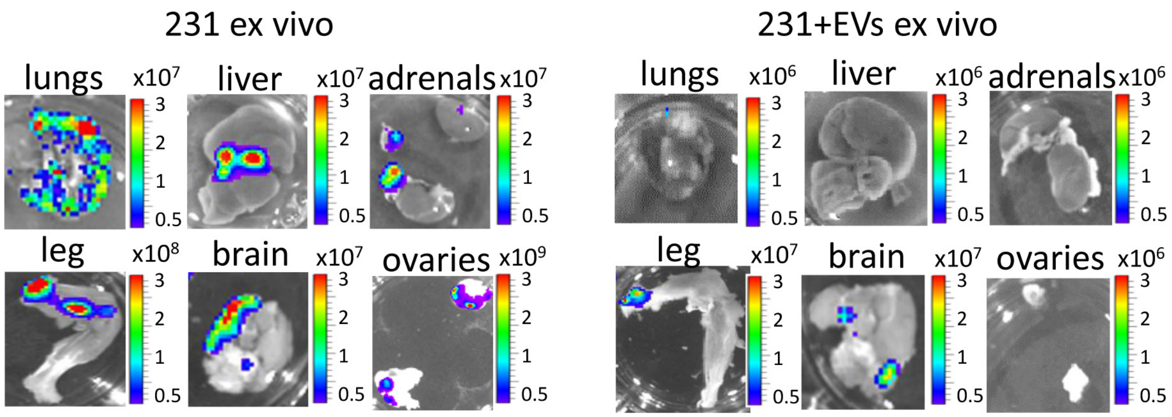

Supplementary Figure 1: 5 × 10^5 MDA-MB-231 cells and hMSC-EV-primed MDA-MB-231 cells were intracardially injected into nude mice. At the endpoint, mice were sacrificed and the total photon of lungs, liver, adrenals, legs, brain, and ovaries were measured, $n = 8$.

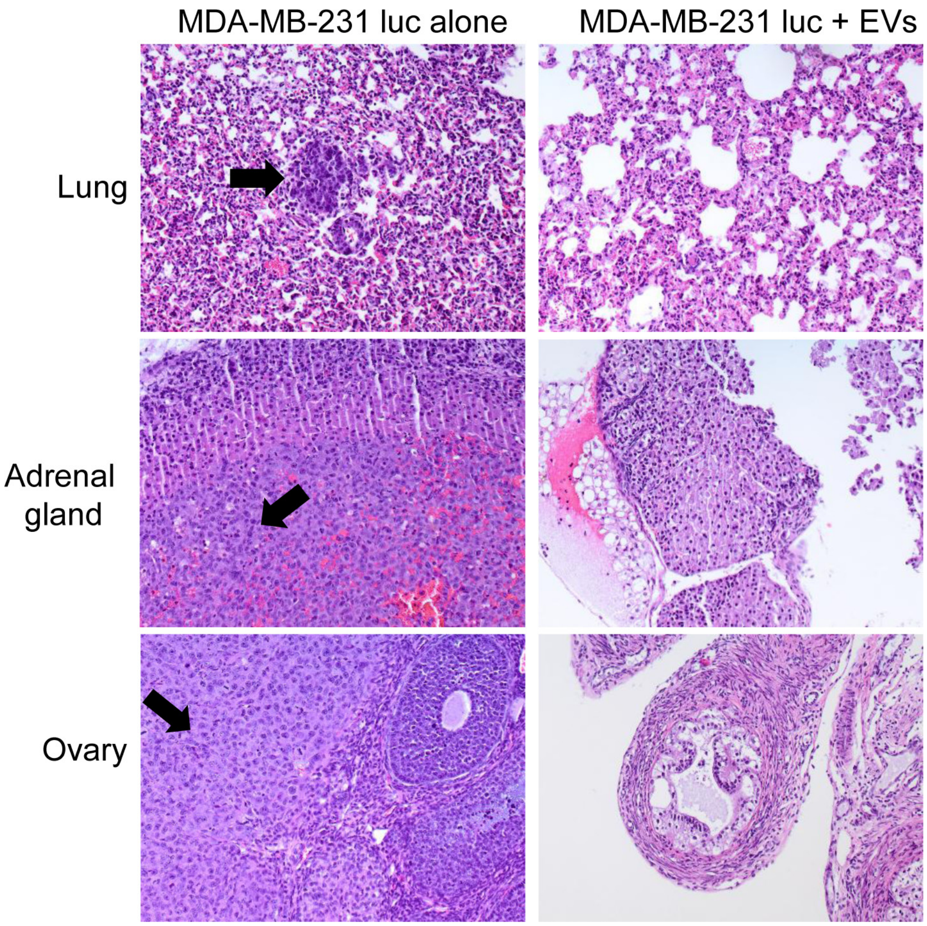

Supplementary Figure 2: Representative images of hematoxylin and eosin staining of tumor sections showing higher grade of tumors (arrows) in mice that received untreated cells compared with the hMSC-s-EV-primed cancer cells.

A

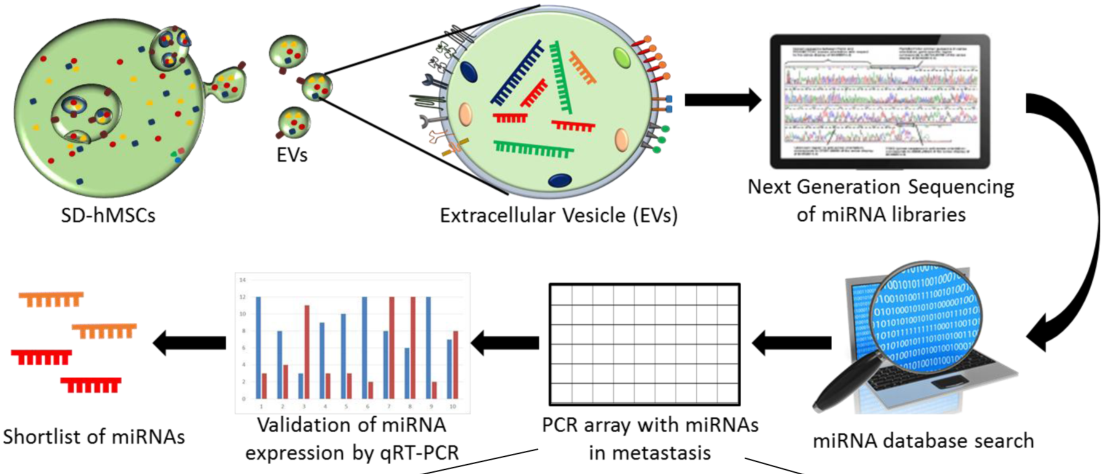

B

miR-30a	miR-100	miR-107	miR-10b	miR-124	miR-1258	miR-125b	miR-126	miR-127	miR-132
miR-143	miR-145	miR-146a	miR-146b	miR-148a	miR-149	miR-183	miR-185	miR-191	miR-193b
miR-195	miR-19a	miR-200c	miR-205	miR-22	miR-26a	miR-26b	miR-31	miR-335	miR-339
miR-34c	miR-379	miR-497	miR-517a	miR-9	miR-99a	5sRNA	18sRNA	RNU6-2	SNORD68

Supplementary Figure 3: (**A**) Schematic overview of the in silico procedures used to shortlist the miRNAs in metastasis. The experimental approach used to validate the shortlisted miRNAs is also depicted. (**B**) The panel of 36 miRNAs that have a known role in metastasis.

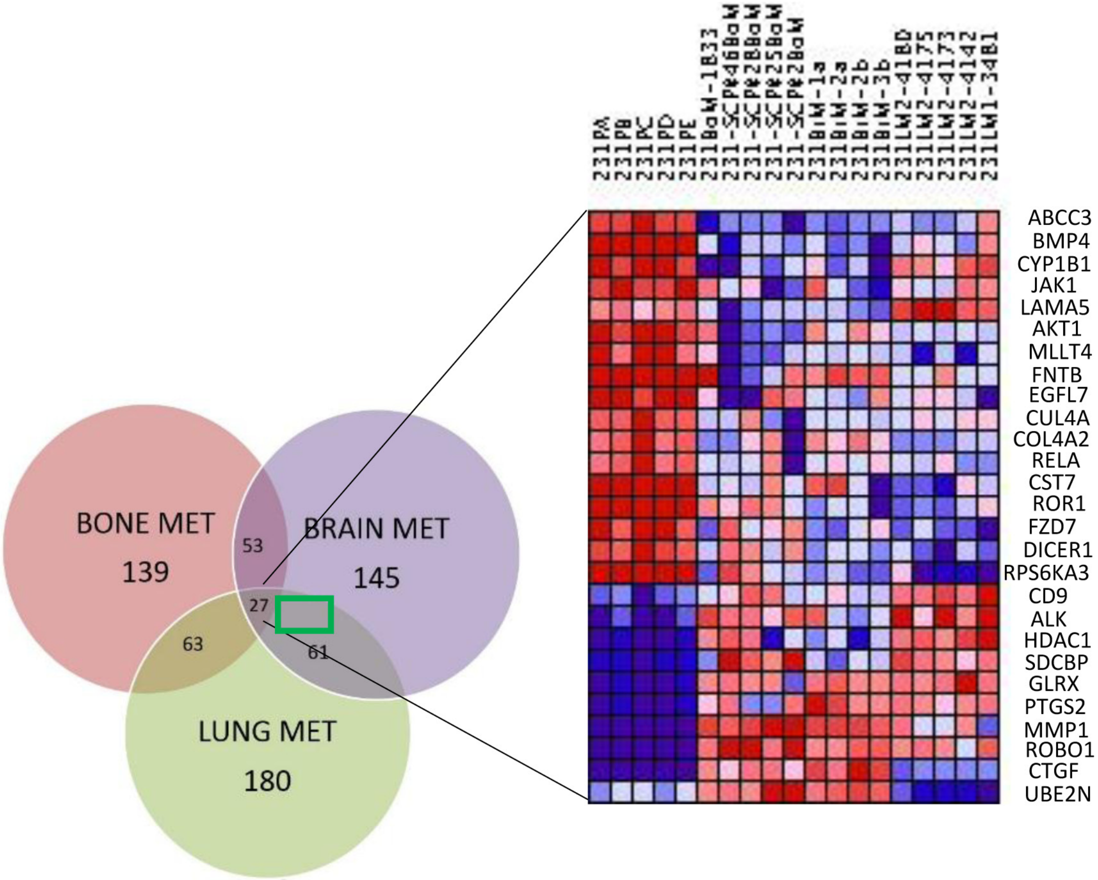

Supplementary Figure 4: Microarray clustering analysis of GEO datasets. A heat map was generated for the 27 genes out of 281 that were significantly up- or downregulated in the metastatic sublines compared with parental cells (MDA-MB-231).

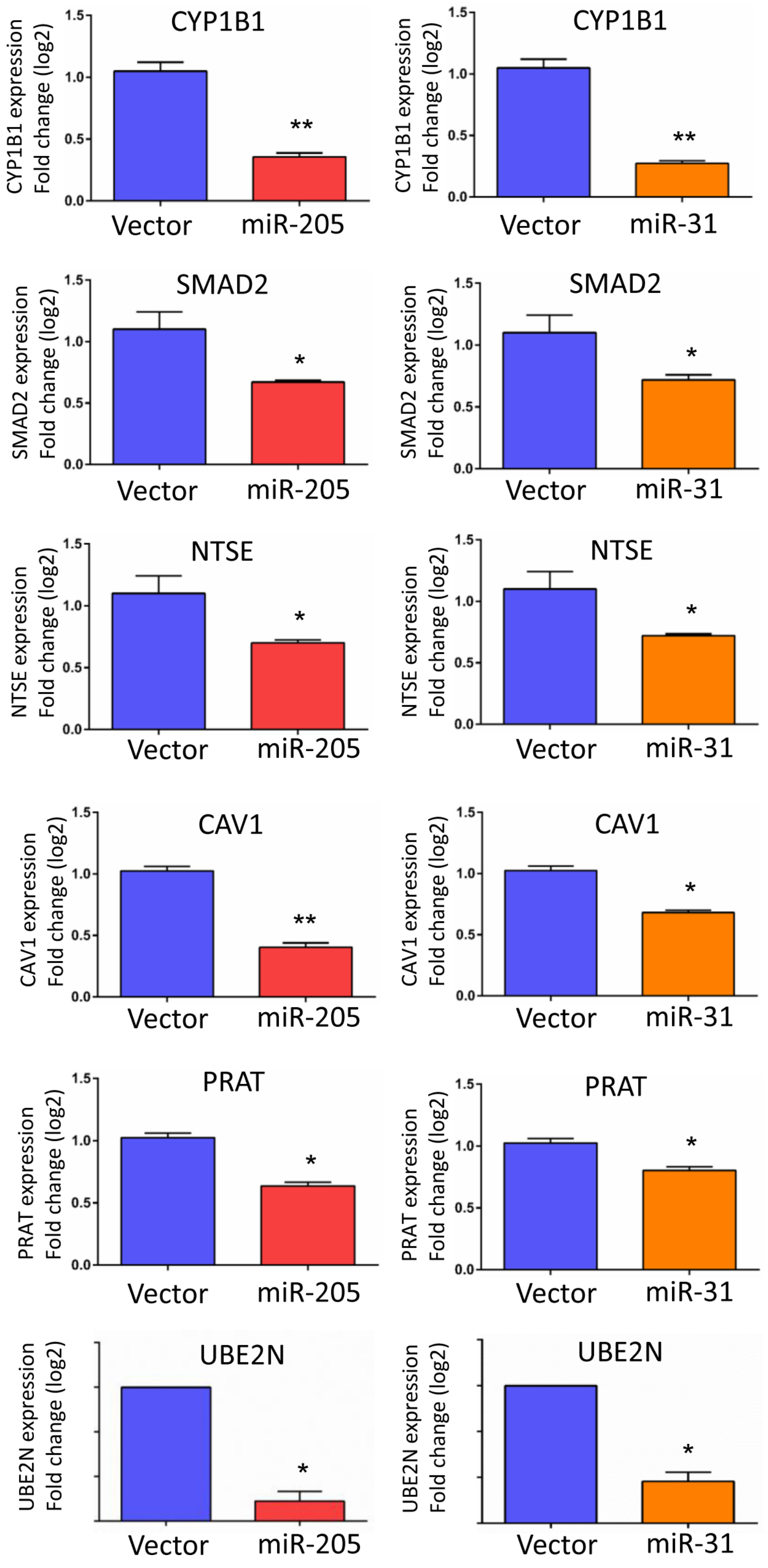

Supplementary Figure 5: Real- time PCR data showing the significant down-regulation of the target genes upon expression of miR-205 and miR-31 by lentiviral transductions.

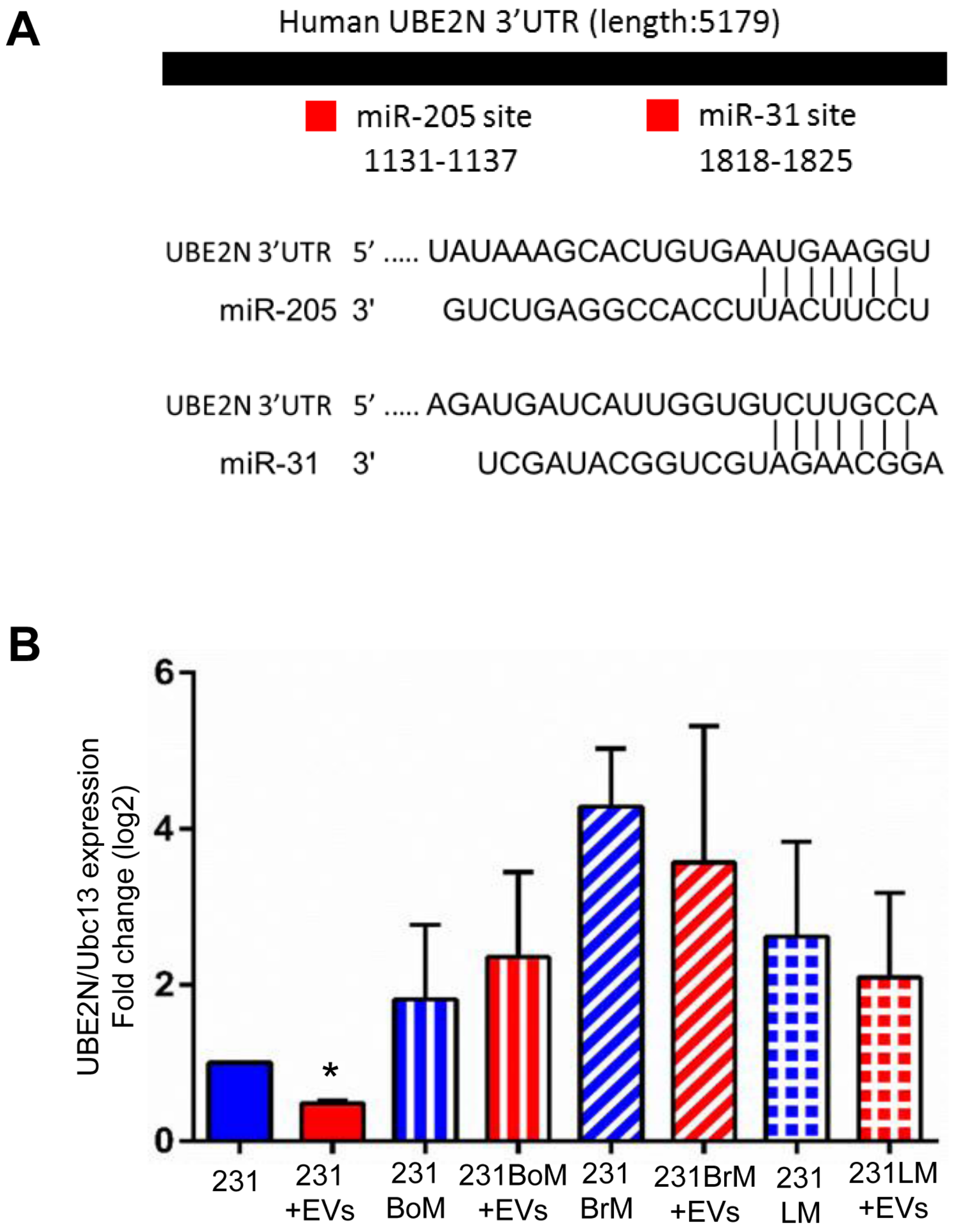

Supplementary Figure 6: (**A**) Schematic representation of the UBE2N 3'-UTR with binding sites for miR-205 and miR-31 and sequence alignment of predicted miR-205 and 31 binding sites on UBE2N 3'-UTR. Complementary sequences of miR-205 and 31 to mammalian UBE2N 3'-UTR are shaded red. (**B**) MDA-MB-231, 231-BM, 231-BrM, and 231-LM cells were treated with hMSC-EVs, and the UBE2N/Ubc13 levels were measured by RT-PCR.
